# Superior haplotypes for haplotype‐based breeding for drought tolerance in pigeonpea (*Cajanus cajan* L.)

**DOI:** 10.1111/pbi.13422

**Published:** 2020-06-22

**Authors:** Pallavi Sinha, Vikas K. Singh, Rachit K. Saxena, Aamir W. Khan, Ragavendran Abbai, Annapurna Chitikineni, Aarthi Desai, Johiruddin Molla, Hari D. Upadhyaya, Arvind Kumar, Rajeev K. Varshney

**Affiliations:** ^1^ Center of Excellence in Genomics & Systems Biology (CEGSB) International Crops Research Institute for the Semi‐Arid Tropics (ICRISAT) Patancheru Telangana State India; ^2^ International Rice Research Institute (IRRI) South‐Asia Hub ICRISAT Campus Patancheru Telangana State India; ^3^ Leibniz Institute of Plant Genetics and Crop Plant Research Gatersleben Germany; ^4^ Ghatal Rabindra Satabarsiki MahaVidyalaya Paschim Medinipur West Bengal India; ^5^ IRRI South Asia Regional Center Varanasi India

**Keywords:** candidate gene‐based association analysis, drought tolerance, haplotype analysis, haplotype‐based breeding, pigeonpea

## Abstract

*Haplotype‐based breedin*g, a recent promising breeding approach to develop tailor‐made crop varieties, deals with identification of superior haplotypes and their deployment in breeding programmes. In this context, whole genome re‐sequencing data of 292 genotypes from pigeonpea reference set were mined to identify the superior haplotypes for 10 drought‐responsive candidate genes. A total of 83, 132 and 60 haplotypes were identified in breeding lines, landraces and wild species, respectively. Candidate gene‐based association analysis of these 10 genes on a subset of 137 accessions of the pigeonpea reference set revealed 23 strong marker‐trait associations (MTAs) in five genes influencing seven drought‐responsive component traits. *Haplo‐pheno* analysis for the strongly associated genes resulted in the identification of most promising haplotypes for three genes regulating five component drought traits. The haplotype *C. cajan_23080*‐H2 for plant weight (PW), fresh weight (FW) and turgid weight (TW), the haplotype *C. cajan_30211‐*H6 for PW, FW, TW and dry weight (DW), the haplotype *C. cajan_26230*‐H11 for FW and DW and the haplotype *C. cajan_26230*‐H5 for relative water content (RWC) were identified as superior haplotypes under drought stress condition. Furthermore, 17 accessions containing superior haplotypes for three drought‐responsive genes were identified. The identified superior haplotypes and the accessions carrying these superior haplotypes will be very useful for deploying *haplotype‐based breeding* to develop next‐generation tailor‐made better drought‐responsive pigeonpea cultivars.

## Introduction

Pigeonpea is an annual crop species, which is generally grown in marginal lands with minimal inputs. Pigeonpea has five broad maturity groups including super early (<90 days), extra‐early (91–120 days), early (121–150 days), medium (161–200 days) and late (>250 days) groups. Most of the traditional varieties grown fall into medium and late maturity groups. The varieties of the medium and late maturity groups are prone to terminal moisture stress which occurs at the pod filling stage. Therefore, breeding efforts were directed towards reducing the maturity time and early, extra‐early and super early varieties were developed to escape the terminal moisture stress. However, due to changes in the rain patterns (long dry spells), almost all the maturity groups suffer from intermittent drought. Moreover, during last few years in India, early drought stages are becoming prevalent where just after sowing or at the seedling stage, crop suffers from drought. Drought stress negatively influences an array of major biochemical and physiological processes leading to a reduction in leaf size, stem elongation, root proliferation, stomatal conductance and water‐use efficiency. Eventually, this leads to a severe decline in yield.

Conventional breeding methods/approaches offered to develop varieties/breeding lines for different ecologies/stresses in many crops. However, it requires much time and additional resources. Genomics‐assisted breeding (GAB) combining sequencing‐based trait mapping and sequencing‐based breeding are changing the way of breeding in many crops (Varshney *et al*., 2019a). Identification of genomic region(s) responsible for the trait of interest is an initial step of crop improvement programme to develop next generation of climate‐smart varieties (Varshney *et al*., 2018). With the advantage of the high‐throughput sequencing and phenotyping technologies, the identification of quantitative trait loci (QTLs)/marker‐trait associations (MTAs) have been accelerated in many crops. Several GAB approaches such as marker‐assisted selection (MAS), marker‐assisted backcrossing (MABC) and marker‐assisted recurrent selection (MARS) have been suggested to transfer/assemble superior alleles into elite genetic background(s). Recently, a 5G (genome, germplasm, gene function, genomic breeding and genome editing) breeding approach has been proposed to bring precision and enhancing breeding efficiency for crop genetic improvement (Varshney *et al*., 2020). Genomic selection (GS) through genomic‐estimated breeding values (GEBVs)‐based prediction breeding approaches have also become popular for crop improvement (Crossa *et al*., 2017; Varshney *et al*., 2012).

With the availability of genome sequence data in recent years, sequencing‐based trait mapping approaches including sequencing of extreme genotypes pools or entire population have been very useful in the identification of QTLs/MTAs in many crops (Varshney *et al*., 2019a). For instance, next‐generation sequencing (NGS)‐based technologies together with precise phenotyping data have been used for identification of marker‐trait associations in rice (Li *et al*., 2014), soybean (Fang *et al*., 2017), chickpea (Varshney *et al*., 2019b), pigeonpea (Varshney *et al*., 2017a) and pearl millet (Varshney *et al*., 2017b). Furthermore, whole genome re‐sequencing (WGRS)‐based association mapping approaches identify marker‐trait associations at higher resolution and a number of haplotypes for identified MTA(s) for the target traits. In this context, Bevan *et al*. (2017) proposed ‘Haplotype assembly’ as one of the promising approaches for developing improved crops in the post‐sequencing era. In recent years, *haplo‐pheno* analysis has been used for identification of superior haplotypes in some crop species. For instance, superior haplotypes of 21 genes governing grain yield and quality traits across 3K rice genomes were identified in our recent study (Abbai *et al*., 2019). Similarly, haplotypes for deep water adaptation (Kuroha *et al*., 2018) and dry direct seeded rice (Chen *et al*., 2019) have been identified. Superior haplotypes of *HKT* family genes contributing to salinity tolerance have been reported upon screening Indian wild rice germplasm (Mishra *et al*., 2016), and superior haplotype for salinity tolerance gene *GamSALT3* (Glycine max salt tolerance‐associated gene on chromosome 3) was reported in soybean (Guan *et al*., 2014).

To understand the molecular mechanisms of drought tolerance in pigeonpea, 51 genes were selected using the Hidden Markov Models (HMMs) having close similarity to universal stress protein domain. Validation of the selected 51 genes was conducted on three pigeonpea genotypes (ICPL 151, ICPL 8755 and ICPL 227) having different levels of drought tolerance. Furthermore, based on gene expression analysis using qRT‐PCR, a set of 10 differentially expressed genes showing ≥ two‐fold up‐regulation in the more drought tolerant genotype was selected. These 10 genes represent plant U‐box protein (four genes), universal stress protein A‐like protein (four genes), cation/H(+) antiporter protein (one gene) and an uncharacterized protein (one gene) (Sinha *et al*., 2016). These genes were analysed in the pigeonpea reference set (292 accessions) for identification of superior haplotypes. Candidate gene‐based association study using the sequencing data of these genes with the drought tolerance phenotyping data on a subset of 137 accessions of the pigeonpea reference set identified 23 MTAs in five genes. Furthermore, superior haplotypes for three of five genes were found to have the potential for developing better drought‐tolerant pigeonpea varieties using *haplotype‐based breeding*.

## Results

### Haplotypes for drought‐responsive genes

Analysis of sequencing data of 292 accessions of pigeonpea reference set with 10 candidate genes provided 925 variants ranging from 23 (*C.cajan_13768*) to 232 (*C.cajan_26230*) (Table S1; S2). While 111 variants were present in the coding regions (missense, silent and non‐sense), the remaining 814 variants were present in noncoding regions. Subsequently, based on these variants, haplotypes were identified, including the heterozygous alleles, ranging from eight (*C.cajan_23080*) to 60 (*C.cajan_30211*) with varying haplotype frequencies across the reference set (Table S2). The frequency of heterozygous haplotypes ranged from 13% (*C.cajan_46779* and *C.cajan_23080*) to 61% (*C.cajan_09181*). The haplotypes with highest and lowest haplotype frequencies were considered as ‘major’ and ‘minor’ haplotypes, respectively. For instance, the major haplotypes H1 for the gene *C.cajan_29830* and H1 for the gene *C.cajan_33874* showed 95.58% frequency (Table S2). The minor haplotypes for all the genes had 0.34% frequency and were represented by only one genotype (Table S2).

### Haplotype diversity in breeding lines, landraces and wild species

A total of 83, 132 and 60 haplotypes were identified for the 10 target genes in breeding lines, landraces and wild species, respectively (Table S3). The number of haplotypes in breeding lines ranged from one (*C.cajan_39705, C.cajan_33874*) to 26 (*C.cajan_30211*). In landraces, haplotypes ranged from three for *C.cajan_13768, C.cajan_23080 and C.cajan_33874* genes to 36 for *C.cajan_26230* gene. In case of wild species, it ranged from one (*C.cajan_29830*) to 7 (*C.cajan_09181, C.cajan_13768, C.cajan_26230, C.cajan_30211, C.cajan_33874, C.cajan_39705 and C.cajan_46779*). It is evident that for majority of genes (60%), maximum number of haplotypes were present in landraces rather than breeding lines or wild species. While in several cases, same haplotypes were present in both landraces and breeding lines, new haplotypes (not present in wild species and landraces) were also identified in breeding lines. For instance, in the case of *C.cajan_13768* gene, wild species had seven haplotypes, but only three of these haplotypes were found in landraces as well as breeding lines. While checking the remaining four haplotypes in wild species, it was found that *C. cajanifolius* had one, *C. scrabaeoides* had two, and *C. platycarpus* had one haplotypes. This indicates that all three of the wild‐type haplotypes present in landraces and breeding lines were derived from *C. scrabaeoides* (Table S3).

Interestingly, out of tested 10 genes, haplotypes of only three genes in cultivated lines showed complete match with either landraces or wild species, while seven genes showed novel haplotypes in breeding lines which are not present in any of the landraces and wild species. The frequency of the novel haplotype of seven genes of breeding lines ranged from 23.07% (*C.cajan_30211*) to 50% (*C.cajan_26230, C.cajan_29830*).

### Phenotyping of the subset panel

**Figure 1:**
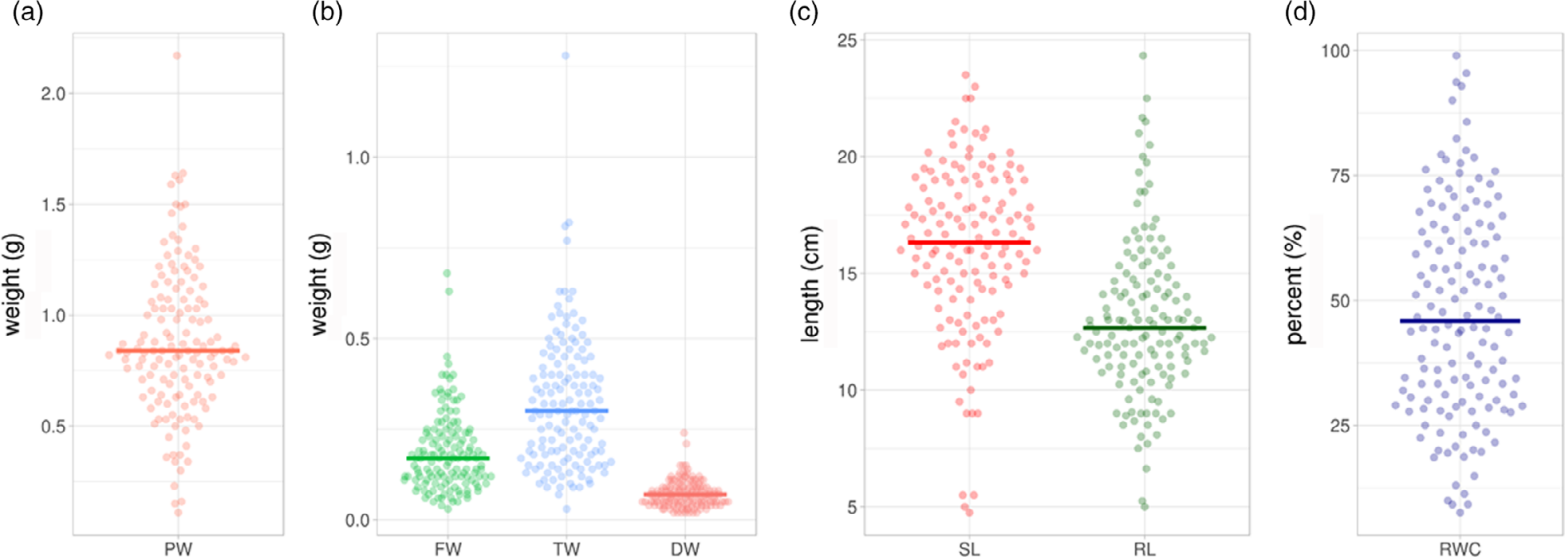
Phenotypic distribution of drought‐responsive traits in 137 diverse accessions of pigeonpea. The subset was phenotyped for plant weight, fresh weight, turgid weight, dry weight and relative water content under drought stress. The violin plots show the phenotypic distribution of the 137 accessions of the pigeonpea reference set for the targeted traits. The shape of the distribution (skinny on each end and wide in the middle) indicates that the trait distribution is highly concentrated around the median except for dry weight (DW). SL—shoot length; RL—root length; FW—fresh weight; DW—dry weight; TW—turgid weight; RWC—relative water content

**Figure 2:**
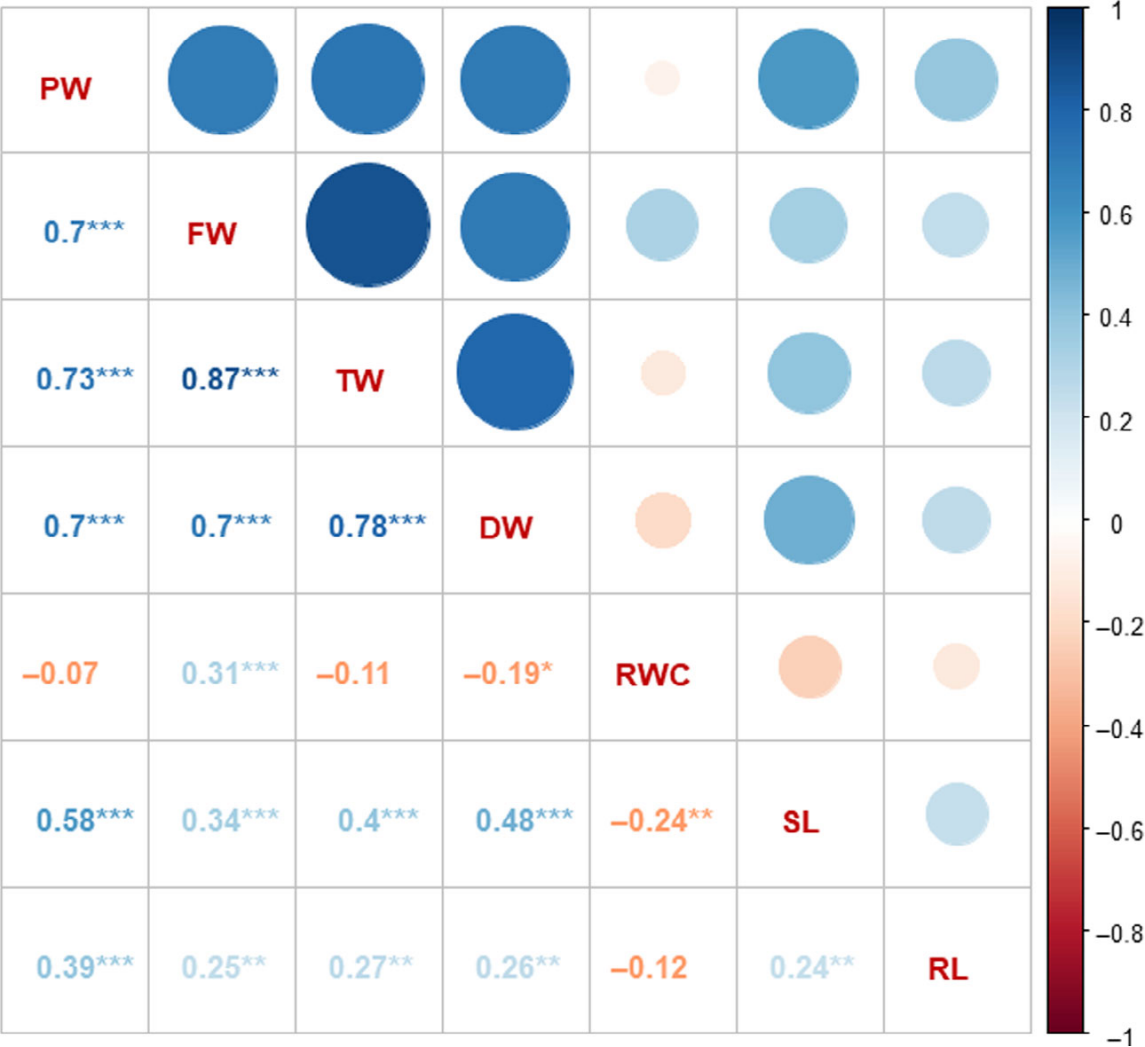
Correlation analysis of the targeted drought‐responsive traits in the phenotyped subset. SL and RL were significantly positively correlated with each other and among the four component traits (PW, FW, TW and DW). The four component traits PW, FW, TW and DW showed a significant positive correlation among each other. DW showed a significant negative correlation, whereas FW showed a significant positive correlation with RWC. SL—shoot length; RL—root length; FW—fresh weight; DW—dry weight; TW—turgid weight; RWC—relative water content. *(*P* < 0.05), **(*P* < 0.01), ***(*P* < 0.001)

Based on detailed analysis, a subset of 137 accessions from the pigeonpea reference set was selected in such a way that these genotypes contained all 232 nonredundant haplotypes identified for all 10 genes. In summary, these 137 accessions include 49 breeding lines, 78 landraces and nine wild species originating from 21 countries. Additionally, there was one genotype present in the subset panel with unknown origin (Table S4). The established subset was phenotyped for plant weight (PW), shoot length (SL), root length (RL), fresh (FW), turgid (TW) and dry weight (DW) of leaves and relative water content (RWC). A significant variation was observed for all the targeted traits in 137 accessions (Figure 1). The PW ranged from 0.11 to 2.17 g (breeding lines: 0.34–1.61 g; landraces: 0.34–2.17 g; wild species: 0.11–0.30 g), SL ranged from 4.75 to 23.50 cm (breeding lines: 9.00–21.17 cm; landraces: 9.00–23.50 cm; wild species: 4.75–9.00 cm), RL ranged from 5.00 to 24.33 cm (breeding lines: 6.63–24.33 cm; landraces: 5.00–21.50 cm; wild species: 9.00–22.50), FW of leaves ranged from 0.03 to 0.68 g (breeding lines: 0.04–0.63 g; landraces: 0.05–0.68 g; wild species: 0.03–0.11 g), TW of leaves ranged from 0.03 to 1.28 g (breeding lines: 0.07–0.63 g; landraces: 0.09–1.28 g; wild species: 0.03–0.35 g) and DW ranged from 0.02 to 0.24 g (breeding lines: 0.02–0.15 g; landraces: 0.02–0.24 g; wild species: 0.02–0.04 g; Table S5). The RWC ranged from 7.58 to 98.96% (breeding lines: 18.63–98.96%; landraces: 7.58–95.45%; wild species: 25.00%–73.33%). This indicated that a significant phenotypic variation was present for the targeted traits in the 137 accessions studied (Figure 1). Correlation analysis was carried out to understand relationships among various drought component traits (Figure 2). SL and RL were significantly positively correlated with each other and with the four component traits (PW, FW, TW and DW). A significant positive correlation was observed with each other for PW, FW, TW and DW. However, DW and FW showed a significant negative and positive correlation with RWC, respectively.

### Association of drought‐responsive genes with phenotype

Candidate gene‐based association analysis using 925 variants in the above‐mentioned 10 drought‐responsive genes and phenotyping data on 137 accessions identified 23 significant MTAs in five genes for the seven component traits of drought (Table 1). It was noted that single gene was associated with more than one component traits. For instance, *C.cajan_30211, C.cajan_23080, C.cajan_26230* and *C.cajan_46779* genes were found to be associated with six, three, two and two drought component traits, respectively. Correlation analysis among seven drought component traits revealed many of the traits were significantly associated with each other and there might be possibility that a gene can be controlling more than one trait.

**Table 1:** Candidate gene‐based association analysis for identification of trait‐associated genes

Trait	Gene	CcLG/Scaffold	SNP position (bp)	Gene (annotation)	*P*‐value	PVE (%)
Shoot length	*C.cajan_30211*	Scaffold126966	344212	U‐box domain‐containing protein 52	0.00490142	4.48
Root length	*C.cajan_30211*	Scaffold126966	349139	U‐box domain‐containing protein 52	0.00129826	8.16
	*C.cajan_29830*	Scaffold128889	297640	Universal stress protein A‐like protein	0.00724983	5.62
Plant weight	*C.cajan_30211*	Scaffold126966	344102	U‐box domain‐containing protein 52	0.00033134	8.85
	*C.cajan_23080*	CcLG05	86455	Universal stress protein	0.00080033	7.67
	*C.cajan_30211*	Scaffold126966	344496	U‐box domain‐containing protein 52	0.00206941	6.42
Fresh weight	*C.cajan_30211*	Scaffold126966	344102	U‐box domain‐containing protein 52	0.00013847	11.41
	*C.cajan_23080*	CcLG05	86455	Universal stress protein	0.00044457	9.61
	*C.cajan_30211*	Scaffold126966	344496	U‐box domain‐containing protein 52	0.00161236	7.67
	*C.cajan_26230*	Scaffold133234	89141	U‐box domain‐containing protein 35	0.00164604	7.64
	*C.cajan_46779*	Scaffold117697	3348	Cation/H(+) antiporter 15	0.0054616	5.9
Dry weight	*C.cajan_26230*	Scaffold133234	91818	U‐box domain‐containing protein 35	4.61E‐06	17.02
	*C.cajan_30211*	Scaffold126966	344102	U‐box domain‐containing protein 52	0.00089919	8.59
	*C.cajan_30211*	Scaffold126966	344496	U‐box domain‐containing protein 52	0.00150988	7.81
Turgid weight	*C.cajan_30211*	Scaffold126966	344102	U‐box domain‐containing protein 52	2.39E‐05	13.62
	*C.cajan_30211*	Scaffold126966	344496	U‐box domain‐containing protein 52	6.70E‐05	12.03
	*C.cajan_23080*	CcLG05	86455	Universal stress protein	0.00026617	9.95
	*C.cajan_46779*	Scaffold117697	3348	Cation/H(+) antiporter 15	0.00142431	7.52
	*C.cajan_46779*	Scaffold117697	1822	Cation/H(+) antiporter 15	0.00395323	6.09
	*C.cajan_30211*	Scaffold126966	345767	U‐box domain‐containing protein 52	0.0053937	5.67
	*C.cajan_30211*	Scaffold126966	345945	U‐box domain‐containing protein 52	0.0053937	5.67
	*C.cajan_30211*	Scaffold126966	348513	U‐box domain‐containing protein 52	0.0053937	5.67
Relative water content	*C.cajan_26230*	Scaffold133234	88787	U‐box domain‐containing protein 35	0.00572797	2.78

CcLG, *Cajanus cajan* linkage group; SNP position, marker‐trait association position with the trait of interest; PVE (%), per cent phenotypic variance explained.

For shoot length (SL), it was found that ‘U‐box domain‐containing protein 52 gene’ (*C.cajan_30211*) was significantly associated with 4.48% phenotypic variance explained (PVE). This gene had 60 haplotypes across the selected subset (as well as the reference set). Interestingly, the same gene also showed association with root length (PVE: 8.16%), plant weight (PVE: 8.85%), fresh weight (PVE: 11.41%), dry weight (PVE: 8.59%) and turgid weight (PVE: 13.62%). Similarly, ‘Universal stress protein’ (*C.cajan_23080*) also showed association with plant weight (PVE: 7.67%), fresh weight (PVE: 9.61%) and dry weight (PVE: 9.95%) at higher level (*P* < 0.01) of statistical significance. This gene had eight haplotypes in the reference set. The ‘U‐box domain‐containing protein 35’ (*C.cajan_26230*) was found associated with control fresh weight (PVE: 7.64%), dry weight (PVE: 17.02%) and RWC (PVE: 2.78%) and had 55 haplotypes. The ‘Cation/H (+) antiporter 15’ gene (*C.cajan_46779*) with 16 haplotypes showed association with fresh weight (PVE: 5.9%) and dry weight (PVE: 7.52%). Finally, the ‘Universal stress protein A‐like protein’ (*C.cajan_29830*) gene was strongly associated with root length (PVE: 5.62%) and was found to possess 13 haplotypes across the selected subset of the pigeonpea reference set.

### Superior haplotypes for drought responsiveness

Haplotype and phenotype (*haplo‐pheno*) analysis identified five strongly associated genes, and the targeted phenotypic traits were utilized to define ‘*superior haplotypes’*. In this analysis, if average phenotypic performance of a group of individuals containing a particular haplotype was significantly higher than the average phenotypic performance of groups of the individuals containing other haplotypes, that particular haplotype has been considered as the superior haplotype. As a result, four superior haplotypes were identified in three genes regulating five traits (Table 2 and Figures S1‐S5). For *C.cajan_23080*, H2 was identified as the superior haplotype which is associated with three drought component traits namely PW, FW and TW. For the gene *C.cajan_26230* two haplotypes, H11 (associated with FW and DW) and H5 (associated with RWC) were identified as the superior haplotype. In the case of *C.cajan_30211*, haplotype H6 was identified as the superior haplotype associated with the traits PW, FW, TW and DW.

**Table 2:** Average performance of accessions possessing superior haplotype in comparison to other group of haplotypes

Trait	Gene	Superior haplotype	Average performance of individuals with superior haplotype	Average performance of individuals with other haplotypes
Plant weight	*C.cajan_30211*	H6	H6‐1.01g^a^	H1‐0.90g^b, c^, H2‐0.79g^c^, H4‐0.83^b^, H5‐0.73g^c^
	*C.cajan_23080*	H2	H2‐1.2g^a^	H1‐0.86g^b^, H3‐0.23^c^, H5‐0.23^c^;
Fresh weight	*C.cajan_30211*	H6	H6‐0.29g^a^	H1‐0.19g^b^, H2‐0.19g^b^, H4‐0.16g^c^, H5‐0.19g^b^
	*C.cajan_23080*	H2	H2‐0.23g^a^	H1‐0.18g^b^, H3‐0.05g^c^, H5‐0.07g^c^
	*C.cajan_26230*	H11	H11‐0.43g^a^	H1‐0.18g^c,d^, H2‐0.15g^e,f^, H3‐0.19^c,d^, H4‐0.17g^d,e^, H5‐0.18g^c,d^, H6‐0.2^c^, H7‐0.2g^c,d^, H9‐0.13^f^, H12‐0.15g^e,f^ H14‐0.11^f^, H17‐0.26g^b^
Turgid weight	*C.cajan_30211*	H6	H6‐0.47g^a^	H1‐0.33g^b^, H2‐0.30g^c^, H4‐0.24g^c^, H5‐0.21g^d^
	*C.cajan_23080*	H2	H2‐0.45g^a^	H1‐0.31g^b^, H3‐0.06g^d^, H5‐0.10g^c^
Dry weight	*C.cajan_26230*	H11	H11‐0.13g^a^	H1‐0.19g^c,d^, H2‐0.05g^c,d^, H3‐0.08g^a,b^, H4‐0.05g^d^, H5‐0.05g^d^, H6‐0.06g^a,b^, H7‐0.08g^b^, H9‐0.07^b,c^, H12‐0.06g^c, d^, H14‐0.09g^a,b^, H17‐0.9g^b^
	*C.cajan_30211*	H6	H6‐0.09g^a^	H1‐0.07g^b^, H2‐0.06g^b^, H4‐0.06g^c^, H5‐0.05g^c^
Relative water content	*C.cajan_26230*	H5	H5‐69.6^a^	H1‐42.31^c^, H3‐42.5^c^, H4‐63.79^a,b^, H6‐53.33^c^, H2‐43.4^c^, H7‐35.89^c,d^, H9‐31.11^d^, H11‐58.3^b^, H12‐42.22^c^, H14‐29.85^d^, H17‐66.1^a,b^

Duncan analysis was employed to test statistical significance at *P* < 0.05. Different alphabets indicate significant differences.

*Haplo‐pheno* analysis of only those haplotype groups was performed in which at least two genotypes were present.

### Identification of accessions carrying superior haplotypes

**Figure 3:**
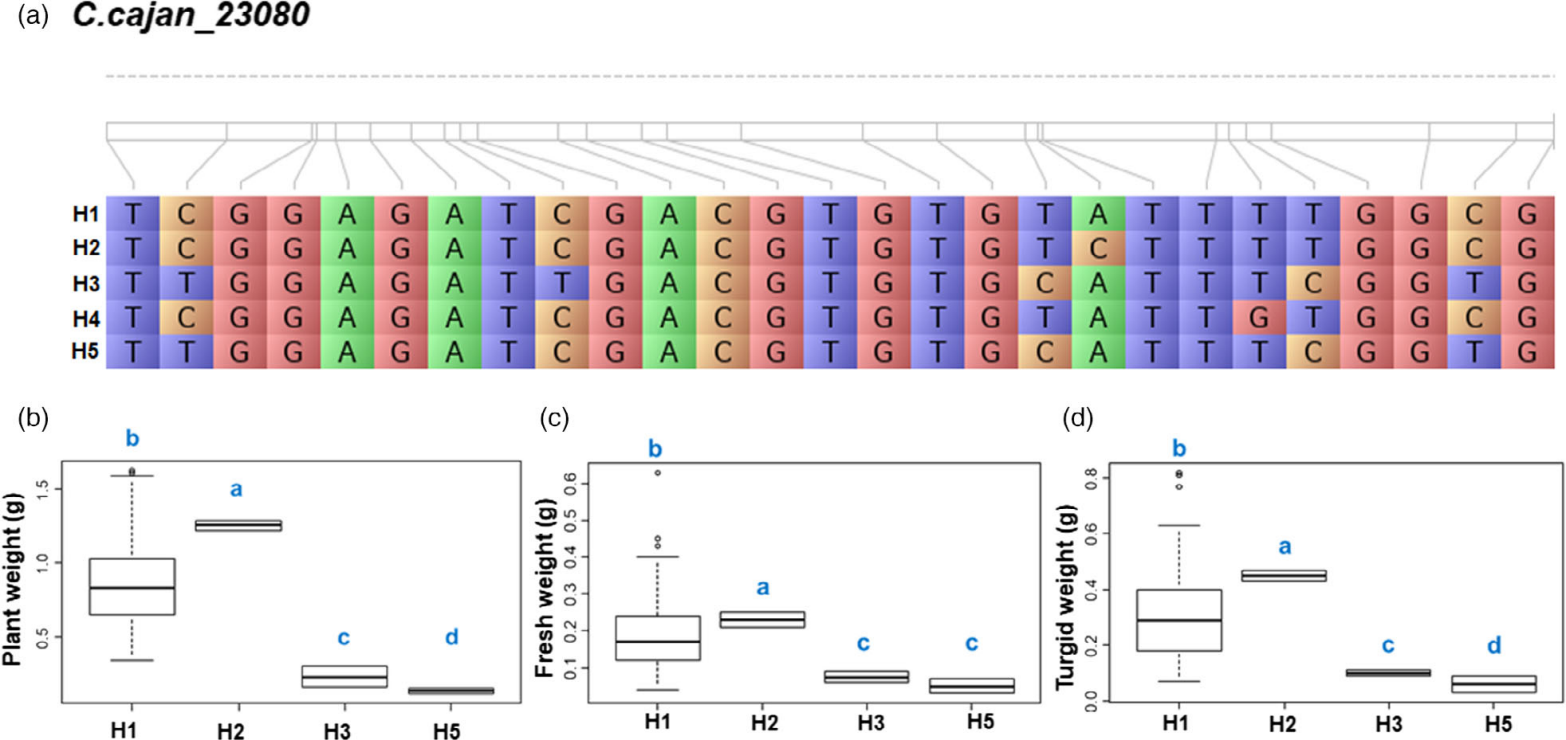
Haplotype analysis of *C.cajan_23080* across the subset panel. (a) Haplotypic variation of *C.cajan_23080*, a gene associated with plant weight, fresh weight and turgid weight. (b) Boxplot showing variation in plant weight, fresh weight and turgid weight among 137 *Cajanus* spp. accessions. Lower and upper boxes indicate the 25th and 75th percentile, respectively. The median is depicted by the horizontal line in the box. Duncan’s analysis suggested H2 is the most superior haplotype of *C.cajan_23080* gene for plant weight, fresh weight and turgid weight

**Figure 4:**
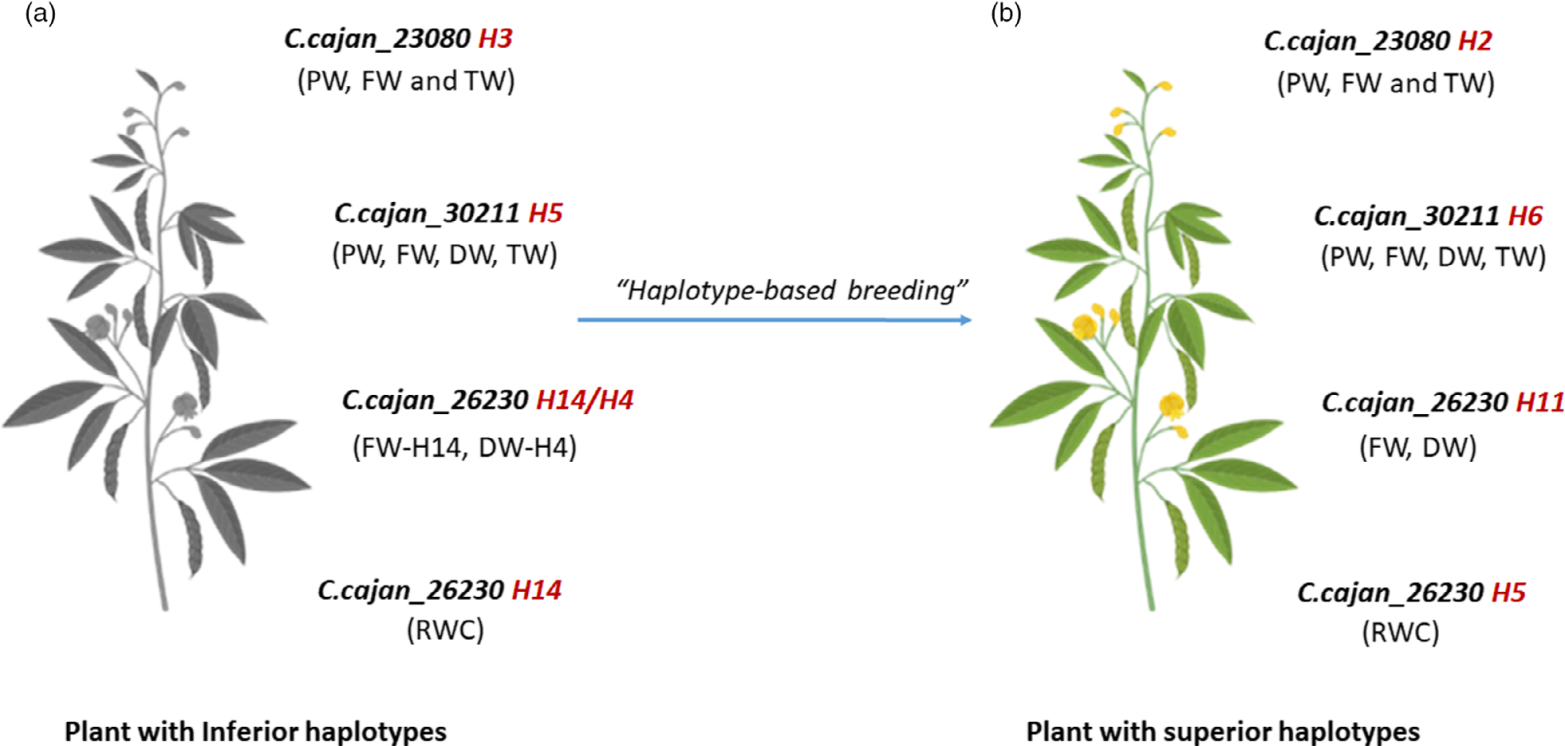
Towards developing tailored pigeonpea with superior haplotypes for drought tolerance. (a) The most inferior haplotype combination for drought responsiveness is *C.cajan_23080*‐H3 (PW, FW and TW), *C.cajan_30211*‐H5 (PW, FW, DW and TW), *C.cajan*_26230‐H14 (FW and RWC) and H4 (DW), and (b) the most superior haplotype combination for enhanced drought responsiveness is *C.cajan_23080*‐H2 (PW, FW and TW), *C.cajan_30211*‐H6 (PW, FW, DW and TW), *C.cajan_26230*‐H11 (FW and DW) and *C.cajan_26230*‐H5 (RWC). Through haplotype‐based breeding, new breeding lines can be developed with the most superior haplotype combination. PW—plant weight; FW—fresh weight; DW—dry weight; TW—turgid weight; RWC—relative water content

A total of 17 accessions (14 landraces and 3 breeding lines) were found carrying superior haplotypes of three genes (*C.cajan_23080*; *C.cajan_30211*; *C.cajan_26230*) associated with the target traits namely PW, FW, TW, DW and RWC (Table 3). For instance, four accessions were found superior for PW (1.2 g), FW (0.23 g) and TW (0.45 g) carrying superior haplotype *C.cajan_23080‐*H2 (Figure 3). Two accessions, ICP 10683 and ICP 7896 harbouring *C.cajan_30211‐*H6 were found superior for four traits namely PW (1.01 g), FW (0.29 g), TW (0.47 g) and DW (0.09 g). Moreover, accessions with *C.cajan_26230*‐H11 had the highest FW (0.43 g) and DW (0.13 g) in drought conditions. Ten accessions with *C.cajan_26230*‐H5 had the highest RWC (69.6%) under drought stress situations (Table 3). Interestingly, an accession was found carrying superior haplotypes for two genes *C.cajan_23080‐*H2 (associated with PW, FW and TW) and *C.cajan_26230*‐H5 (associated with RWC). The identified superior haplotypes governing the major drought component traits (PW, FW, DW, TW and RWC) in the current study (Figure 4) are expected to be useful in the development of next‐generation drought‐tolerant pigeonpea cultivars through *haplotype‐based breeding*.

**Table 3:** List of accessions carrying superior haplotypes for three drought‐associated responsive genes

Genotype	Gene(s)	Superior haplotypes					Biological status	Region	Geographic origin (country)
		PW	FW	TW	DW	RWC			
ICP 10447	*C.cajan_23080*	H2	H2	H2			Landrace	South Asia	India
ICP 1156	*C.cajan_23080*	H2	H2	H2			Landrace	South Asia	India
ICP 1273	*C.cajan_23080*	H2	H2	H2			Landrace	South Asia	India
	*C.cajan_26230*					H5	Landrace	South America	Venezuela
ICP 9236	*C.cajan_23080*	H2	H2	H2			Breeding line	South Asia	India
ICP 10683	*C.cajan_30211*	H6	H6	H6	H6		Breeding line	South Asia	India
ICP 7896	*C.cajan_30211*	H6	H6	H6	H6		Landrace	South Asia	India
ICP 12765	*C.cajan_26230*		H11		H11		Landrace	South Asia	Philippines
ICP 14163	*C.cajan_26230*		H11		H11		Landrace	South Asia	Indonesia
ICP 12410	*C.cajan_26230*					H5	Landrace	Unknown	Unknown
ICP 13191	*C.cajan_26230*					H5	Landrace	South Asia	India
ICP 14971	*C.cajan_26230*					H5	Landrace	South Asia	Indonesia
ICP 2698	*C.cajan_26230*					H5	Landrace	South Asia	India
ICP 4167	*C.cajan_26230*					H5	Landrace	South Asia	India
ICP 6992	*C.cajan_26230*					H5	Landrace	South Asia	India
ICP 7420	*C.cajan_26230*					H5	Landrace	South Asia	India
ICP 8012	*C.cajan_26230*					H5	Landrace	South Asia	India
ICP 7314	*C.cajan_26230*					H5	Breeding line	South Asia	India

DW, dry weight; FW, fresh weight; PW, plant weight; RWC, relative water content; TW, turgid weight.

## Discussion

The concept of haplotype analysis is of great significance as it enables extensive utilization of available genetic variation among the target genes. For instance, *GmCHX1* was identified as the potential candidate conferring salinity tolerance in soybean. Also, the genotypes belonging to SV‐2 haplotype of *GmCHX1* were found to be highly tolerant (Patil *et al*., 2016). Similarly, haplotype analysis for grain cooking and eating quality traits in rice resulted in the identification of superior and desired haplotypes associated with the trait (Wang *et al*., 2017). Recently, haplotype analysis of 120 genes across the 3K panel was conducted to facilitate tailored rice development (Abbai *et al*., 2019). Similarly, haplotypes of five potential candidate genes suitable for dry direct seeded rice were uncovered (Chen *et al*., 2019).

The 292 diverse accessions reported in our earlier study were utilized for harnessing haplotype diversity of target genes related to drought tolerance (Varshney *et al*., 2017a). For this purpose, previously known drought‐responsive candidate genes were selected for the haplotype analysis (Sinha *et al*., 2016). Considering the complexity of drought tolerance, an approach involving PEG phenotyping and candidate gene‐based association analysis revealed strongly associated drought‐tolerant genes in pigeonpea. Correlation analysis among seven drought component traits revealed that many of the traits are significantly associated with each other and there might be possibility that a gene can be controlling more than one trait. Haplotype analysis revealed rich diversity for these genes across the reference set. Further, significant variations were found for all of the seven major drought tolerance‐related traits among the subset. Eventually, superior haplotypes were identified for about five drought influencing components including PW, FE, TW, DW and RWC.

Haplotypic variation of a given region depends on evolutionary and population genetic factors such as mutation and recombination rates and selection (Zaitlen *et al*., 2005). Therefore, to capture the entire haplotypic variation, we also included the heterozygous haplotypes in analysing the haplotype diversity. Interestingly, in the current study, it was observed that more than 80% of haplotype diversity from landraces has already been utilized in breeding lines for two genes (*C.cajan_08737* and *C.cajan_13768*), whereas less than 20% of diversity has been harnessed for the remaining eight genes. This directly sheds light on the fact that only limited haplotype diversity is being utilized in the past and ongoing pigeonpea breeding programmes. Identified superior haplotypes were mostly found in the landraces compared to the breeding lines. This trend suggests their role in the development of drought tolerant breeding lines. Results also showed that the superior haplotypes of *C.cajan_23080* (H2), *C.cajan_30211* (H6) and *C.cajan_26230* (H5) exist in the breeding lines ICP 9236, ICP 10683 and ICP 7314, respectively, were transferred from landraces (Table 3 and Table S3). However, no superior haplotype was identified in the seven accessions of the three wild species utilized in the present study. Moreover, it might be difficult to identify superior haplotypes directly from wild species due to the latent effect of genes and therefore requires the transfer of different haplotypes into elite backgrounds to understand their real effect on the phenotype. Wild species and landraces are the source of potential genes governing important traits, including yield and stress responsiveness. For instance, QTL for tomato fruit size (Frary *et al*., 2000), grain yield in rice (Swamy and Sarla, 2008), etc., are few of the classical case studies where the potential of wild species is deciphered. Besides, wild lentil species and wild chickpeas were found to adapt in drought‐prone areas by reprogramming transpiration rates (Zhang *et al*., 2019). Further, this scenario suggests that more efforts are to be made in the coming years to utilize the existing haplotype diversity for improving drought tolerance in pigeonpea.

Several crop ideotypes have been developed including rice (Khush, 1995) and wheat (Semenov and Stratonovitch, 2013), and the currently proposed strategy shall be precisely utilized towards the development of drought‐tolerant ideotype, sustain pigeonpea varieties with good yield potential. In this context, the results obtained in the current study will not only be used in breeding for drought tolerance, but with the availability of WGRS and phenotypic data of 292 accessions, this can also be utilized towards several component traits. Ideotype breeding plays an important role in shaping the plant architecture, for example together with drought component traits, other crucial component traits of plants, that is days to flowering, plant height, number of branches, number of pods per plant, determinant and in‐determinant types (based on the requirements) and disease resistance (fusarium wilt and sterility mosaic disease).

In this context, a wide range of haplotype‐specific responses to drought was witnessed for all the component traits. Accessions with haplotypes *C.cajan_23080*‐H2 and *C.cajan_30211*‐H6 had the lowest PW under drought, while *C.cajan_23080*‐H2 and *C.cajan_30211*‐H6 were the highest PW. Similarly, for FW, accessions with haplotypes *C.cajan_23080*‐H2, *C.cajan_30211*‐H6 and *C.cajan_26230*‐H11 had the highest and *C.cajan_23080*‐H3, *C.cajan_30211*‐H4 and *C.cajan_26230*‐H14 had the lowest FW‐associated haplotypes. In the case of RWC, accessions with haplotype *C.cajan_26230*‐H5 had the highest, and on the other hand, *C.cajan_26230*‐H9 was with the lowest haplotype for RWC. The superior‐most haplotype combination for enhanced drought responsiveness is *C.cajan_23080*‐H2 (PW, FW and TW), *C.cajan_30211*‐H6 (PW, FW, TW and DW), *C.cajan*_*26230*‐H11 (FW) and *C.cajan_26230‐*H5 (RWC). A total of 17 accessions with superior haplotypes of targeted genes were mostly identified in landraces (14 accessions) as compared to breeding lines (3), indicating that a very less haplotype diversity is utilized in drought breeding programmes. Accessions carrying superior haplotypes were analysed, and interestingly, *C.cajan_26230*‐H11 was present only in landraces, while *C.cajan_23080*‐H2 and *C.cajan_30211‐*H6 and *C.cajan_26230*‐H5 were present in both landraces and breeding lines. No superior haplotype was identified for SL and RL in the study. It is noteworthy that among the selected set of genotypes for haplotype analysis, the two leading varieties, ICPL 8863 and ICPL 151, do not possess any superior haplotype for drought responsiveness. Hence, these varieties can be further improved or better drought‐tolerant varieties could be developed using *haplotype‐based breeding* strategy (Varshney *et al*., 2020).

This *haplotype‐based breeding* strategy shall also be very useful in pigeonpea hybrid breeding, mainly in the selection of parents based on the presence of superior and diverse haplotypes. The parental lines with a set of diverse haplotypes may be best suited for the development of the next generation of superior haplotypes. In this context, parental lines need to be genotyped with the haplotype targeted markers for the development of *haplotype‐based map* (Hap‐Map) of the parental lines, which shall be later on utilized in the breeding programmes. If the superior haplotypic combinations are not present in the parental lines, new parental lines shall be developed through *haplotype‐based breeding* with desired haplotypes. However, a better understanding of the interaction of different haplotypes of different genes controlling the targeted traits shall be studied.

## Conclusions

Haplotype diversity of the potential drought‐responsive genes was harnessed across the pigeonpea reference set. Seven component traits that influence drought, such as plant weight, shoot and root length, fresh, turgid and dry weight, and relative water content, were phenotyped across the chosen diverse subset. Candidate gene‐based association analysis revealed 23 significant marker‐trait associations across five genes. Importantly, superior haplotypes were identified for *C.cajan_23080*‐H2 (PW, FW and TW), *C.cajan_30211*‐H6 (PW, FW, TW and DW), *C.cajan_26230*‐H11 (FW and DW) and *C.cajan_26230*‐H5 (RWC). We expect that in future, further functional evaluation, including uncovering epistatic interactions of these haplotypes and the implementation of *haplotype‐based breeding,* would lead to the development of drought‐tolerant pigeonpea varieties.

## Materials and methods

### Plant material

A set of 292 pigeonpea reference set (including 117 breeding lines, 166 landraces, 2 others and 7 genotypes from three wild species) was used for haplotype analysis of the selected 10 drought‐responsive genes. Further, a subset of 137 diverse accessions from the reference set, representing at least one haplotype of the ten drought‐responsive genes, was selected for validation of the identified haplotypes (Table S4).

### Drought‐tolerance phenotyping

Seeds of the selected 137 accessions were germinated in 3‐inche plastic pots (three seeds per pot) filled with autoclaved black soil, sand and vermicompost (10:10:1 v/v) mixture. Seedlings were grown in controlled glasshouse conditions (25–30°C and ~70% relative humidity) under normal daylight condition. Stress was imposed on 30‐day‐old seedlings of selected 137 accessions using 40% (w/v) PEG6000 for six days. A measured amount of PEG6000 solution was poured every alternate day to maintain uniform stress conditions throughout the experiment. On the seventh day of stress imposition, phenotypic trait was recorded.

Plants were grown in three replications, and the average of 10 seedlings was used for each replicate to generate mean plant weight (FW, g), root length (RL, cm) and shoot length (SL, cm).

To determine relative water content (RWC), ten leaves from each group were weighed immediately to take fresh weight (FW, g) after harvesting the plant. Leaves were then placed in distilled water for 4 h, and then, turgid weight (TW, g) was measured. After this, the leaves were dried in an oven at 80^ º^C for 24hr to obtain their dry weight (DW, g). The following formula calculated relative water content:

$$\text{RWC}\;=\;\left(\text{FW - DW/TW - DW}\right)\times 100.$$

### Haplotype analysis

In our earlier study, 10 drought stress‐responsive candidate genes representing plant U‐box protein (four genes), universal stress protein A‐like protein (four genes), cation/H(+) antiporter protein (one gene) and an uncharacterized protein (one gene) were identified showing expression variation on parents of mapping populations (ICPL 151, ICPL 8755 and ICPL 227) segregating for drought tolerance (Table S1). For haplotype analysis, full‐length sequences of the 10 genes were downloaded from 292 pigeonpea whole‐genome re‐sequencing data using an in‐house script (Varshney *et al*., 2017a). The downloaded sequences were mapped and aligned to the pigeonpea reference genome to find out the variants among 292 accessions using SAM tools (Li *et al*., 2009). The identified variants were later utilized for haplotype analysis using Haploview software (Barrett *et al*., 2005).

### Candidate gene‐based association analysis

The SNPs’ variation underlying the selected 10 candidate genes were used for SNP‐based association analysis. A mixed linear model (MLM) considering genetic relationships or matrix kinship (K) and population structure (Q) in GAPIT was utilized to perform candidate gene‐based association analysis of 10 genes. Marker‐trait associations with *P*‐value < 0.01 were considered significant. Further, superior haplotypes were identified for the strongly associated genes with the traits.

### *Haplo‐pheno* analysis

To associate identified haplotypes of the selected genes with the superior drought tolerance phenotype, a haplo‐pheno analysis was performed. In this regard, first of all, the haplotype present in only one genotype was removed from the analysis. Further, the genotypes were categorized based on haplotype groups, and together with phenotypic data, the superior haplotypes were identified. Haplotype‐wise means of the corresponding traits viz., SL, RL, PW, FW, DW, TW and RWC were compared to define superior haplotypes. Duncan analysis was employed to test statistical significance among the mean of haplotype groups. Different alphabets indicated in the graphs revealed significant differences between the groups at *P* < 0.05 level of significance.

## Conflict of interest

The author(s) declare that they have no competing interests.

## Author contributions

RKV conceived the idea and supervised the study. PS performed most of the analysis. PS, VKS, RKS, AK and RKV interpreted the results. PS, VKS, RKS and RKV wrote the manuscript. AC and RKS contributed in sequence data generation. PS, VKS, AWK and RA carried out statistical analysis. HDU provided the genetic material. PS, AD and JM generated and analysed phenotypic data. All authors read and approved the final manuscript.

## Supporting information


** **

** **

** **

**Figure S1**. Boxplot showing variation in plant weight among 137 *Cajanus* spp. accessions with different haplotypes for identification of superior haplotype for plant weight.
**Figure S2**. Boxplot showing variation in fresh weight among 137 Cajanus spp. accessions with different haplotypes for identification of superior haplotype for plant weight.
**Figure S3**. Boxplot showing variation in turgid weight among 137 Cajanus spp. accessions with different haplotypes for identification of superior haplotype for plant weight.
**Figure S4**. Boxplot showing variation in dry weight among 137 Cajanus spp. accessions with different haplotypes for identification of superior haplotype for plant weight.
**Figure S5**. Boxplot showing variation in relative water content (RWC) among 137 Cajanus spp. accessions with different haplotypes for identification of superior haplotype for plant weight.
**Table S1**. List of genes selected for haplotype analysis.
**Table S2**. Haplotype frequency of 10 drought‐responsive genes.
**Table S3**. Number of unique haplotypes, distribution and frequency range of 10 selected genes in 292 pigeonpea reference set.
**Table S4**. Details of 137 Cajanus spp. accessions utilized for Haplo‐pheno analysis.
**Table S5**. Descriptive statistics of 137 Cajanus spp. accessions subset of reference lines of the targeted drought responsive traits.
