# Supplementary material for: Superior haplotypes for haplotype‐based breeding for drought tolerance in pigeonpea (Cajanus cajan L.)
Source: Plant Biotechnol J. 2020 Jun 22;18(12):2482–90. doi: 10.1111/pbi.13422 (PMC7680530; doi:10.1111/pbi.13422)
Supplement: Supplementary file 2 — Figure S1. Boxplot showing variation in plant weight among 137 Cajanus spp. accessions with different haplotypes for identification of superior haplotype for plant weight. Figure S2. Boxplot showing variation in fresh weight among 137 Cajanus spp. accessions with different haplotypes for identification of superior haplotype for plant weight. Figure S3. Boxplot showing variation in turgid weight among 137 Cajanus spp. accessions with different haplotypes for identification of superior haplotype for plant weight. Figure S4. Boxplot showing variation in dry weight among 137 Cajanus spp. accessions with different haplotypes for identification of superior haplotype for plant weight. Figure S5. Boxplot showing variation in relative water content (RWC) among 137 Cajanus spp. accessions with different haplotypes for identification of superior haplotype for plant weight. Table S1. List of genes selected for haplotype analysis. Table S5. Descriptive statistics of 137 Cajanus spp. accessions subset of reference lines of the targeted drought responsive traits. [file PBI-18-2482-s002.docx]

**Superior haplotypes for drought tolerance in pigeonpea (*Cajanus cajan* L.)**

Pallavi Sinha^1^, Vikas K Singh^2^, Rachit K Saxena^1^, Aamir W Khan^1^, Ragavendran Abbai^2,3^, Annapurna Chitikineni^1^, Aarthi Desai^1^, Johiruddin Molla^1,4^, Hari D. Upadhyaya^1^, Arvind Kumar^2,5^, Rajeev K Varshney^1,^*

**Running title**

Haplotypes for drought tolerance

^1^Center of Excellence in Genomics & Systems Biology (CEGSB), International Crops Research Institute for the Semi-Arid Tropics (ICRISAT), Patancheru- 502324, Telangana State, India

^2^International Rice Research Institute (IRRI), South-Asia Hub, ICRISAT Campus, Patancheru- 502324, Telangana State, India

^3^Leibniz Institute of Plant Genetics and Crop Plant Research, Gatersleben- 06466, Germany

^4^Ghatal Rabindra Satabarsiki Mahavidyalay, West Bengal- 721212, India

^5^IRRI South Asia Regional Center, NSRTC Campus, G.T. Road, Collectry Farm, P.O. Industrial Estate, Varanasi-221 006, India

**^*^Author for Correspondence**

Rajeev K Varshney

International Crops Research Institute for the Semi-Arid Tropics (ICRISAT)

Patancheru - 502 324, India

Telephone: 91-40-30713305;

Fax: 91-40-30713074

E-mail: [r.k.varshney@cgiar.org](mailto:r.k.varshney@cgiar.org)

**Short Summary:** Drought responsive genes have been used for haplotype analysis across 292 genotypes of the pigeonpea reference set. Superior haplotypes for five candidate genes associated with seven major drought component traits have been identified in the present study. Identified favorable haplotypes provide opportunities for developing drought tolerant and climate resilient pigeonpea varieties.


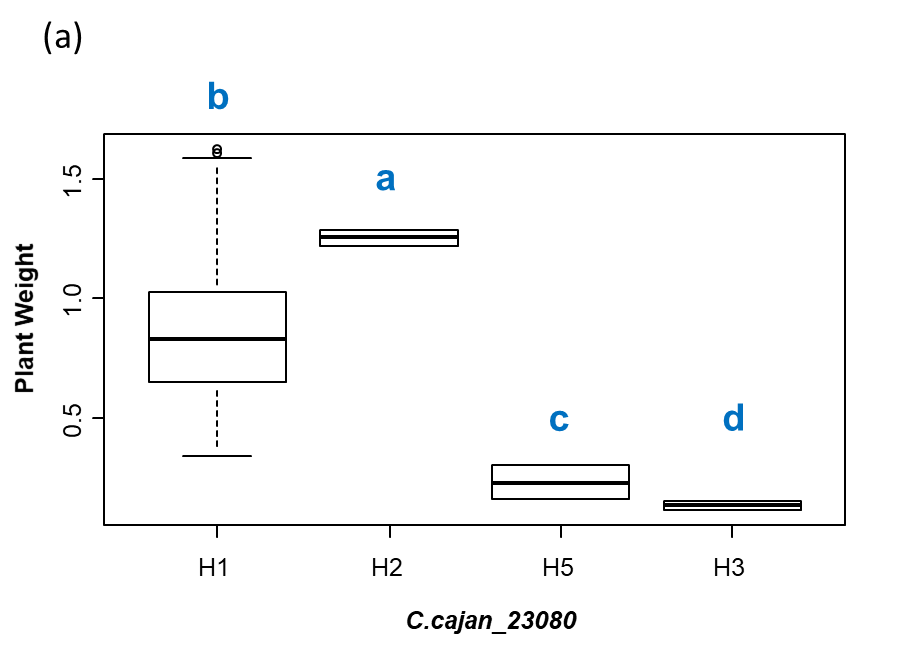


**
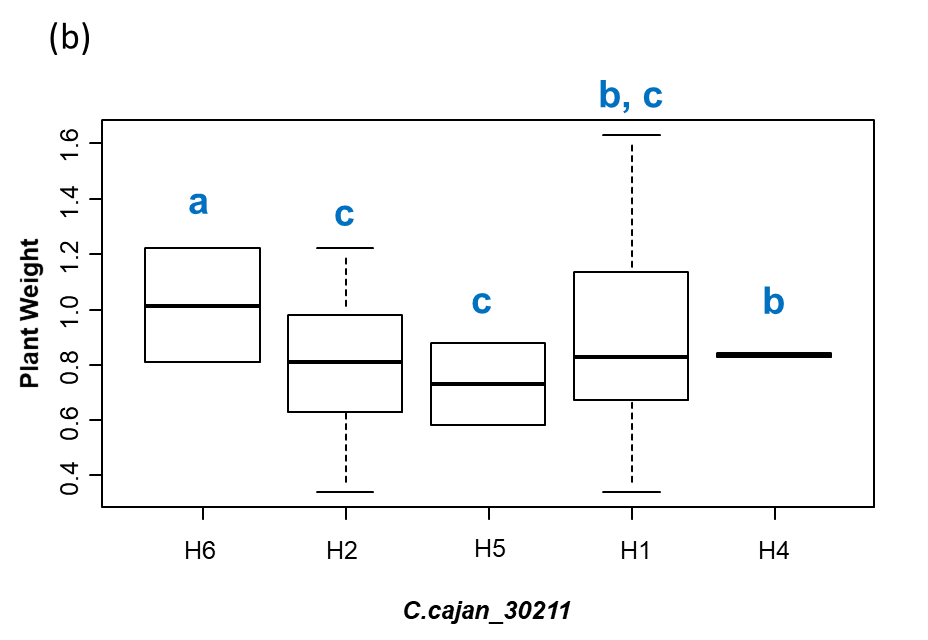
**

**Figure S1.** Boxplot showing variation in plant weight among 137 pigeonpea accessions with different haplotypes for identification of superior haplotype for plant weight. Lower and upper boxes indicate the 25th and 75th percentile, respectively. The median is depicted by the horizontal line in the box. Duncan’s analysis suggested (a) H2 is superior haplotype for *C.cajan_23080* and (b) H6 is superior haplotype for *C.cajan_30211.*


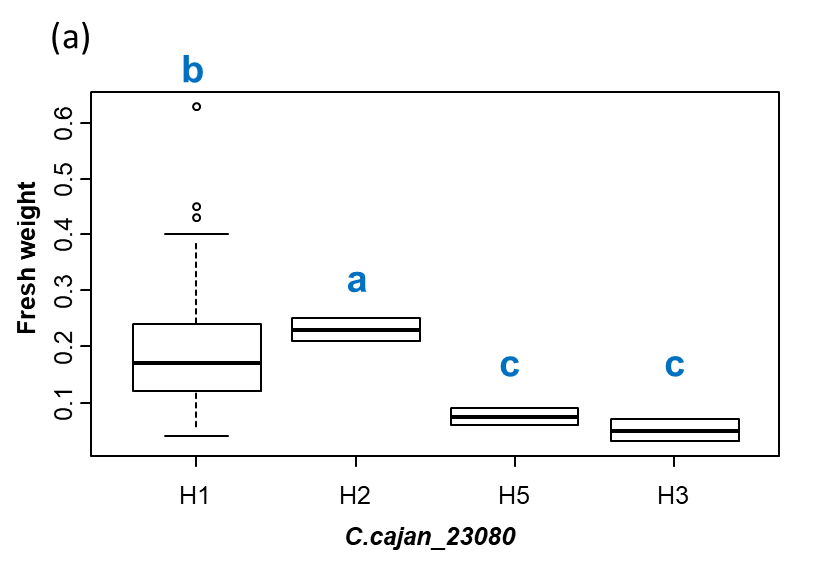


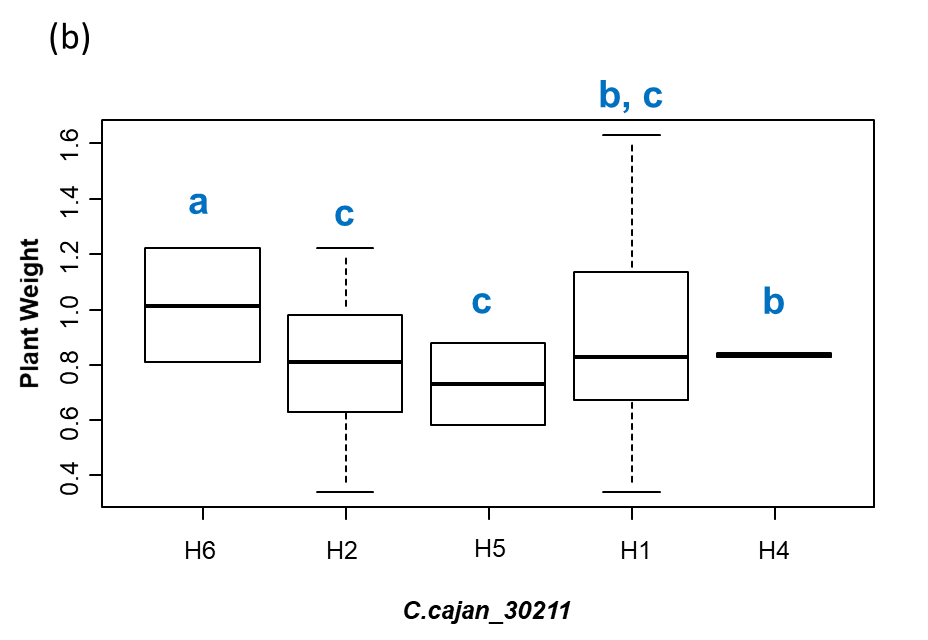


**Figure S2.** Boxplot showing variation in fresh weight among 137 pigeonpea accessions with different haplotypes for identification of superior haplotype for plant weight. Lower and upper boxes indicate the 25th and 75th percentile, respectively. The median is depicted by the horizontal line in the box. Duncan’s analysis suggested (a) H2 is superior haplotype for *C.cajan_23080* (b) H6 is superior haplotype for *C.cajan_30211*


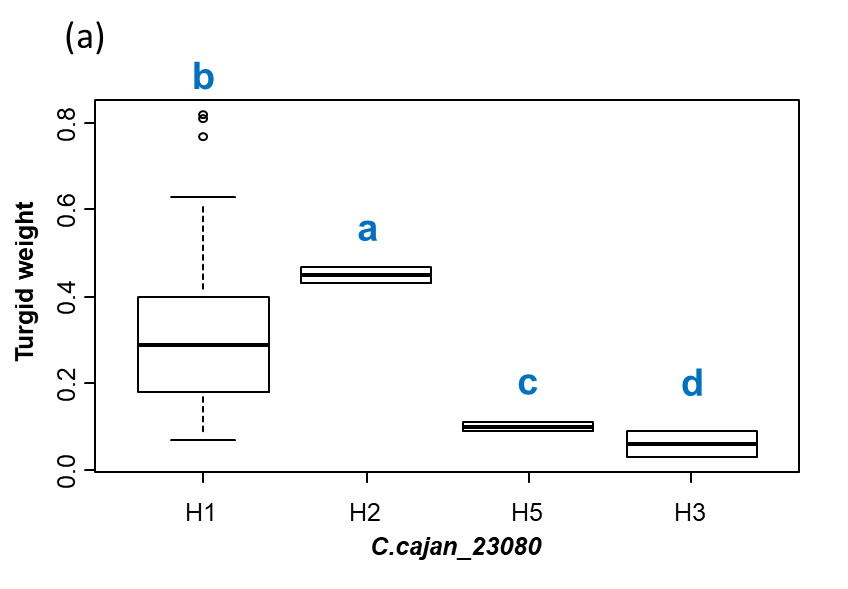


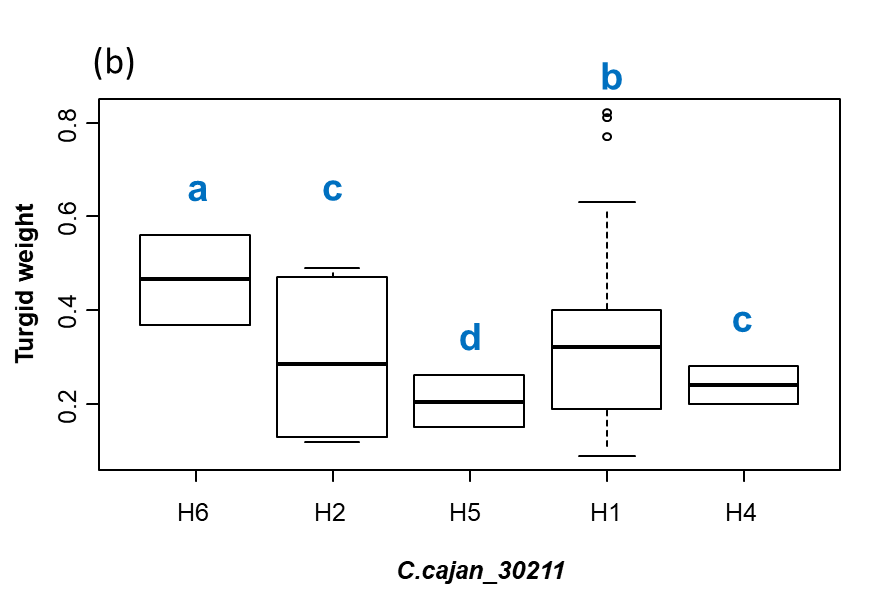


**Figure S3.** Boxplot showing variation in turgid weight among 137 pigeonpea accessions with different haplotypes for identification of superior haplotype for plant weight. Lower and upper boxes indicate the 25th and 75th percentile, respectively. The median is depicted by the horizontal line in the box. Duncan’s analysis suggested (a) H2 is superior haplotype for *C.cajan_23080* (b) H6 is superior haplotype for *C.cajan_30211*


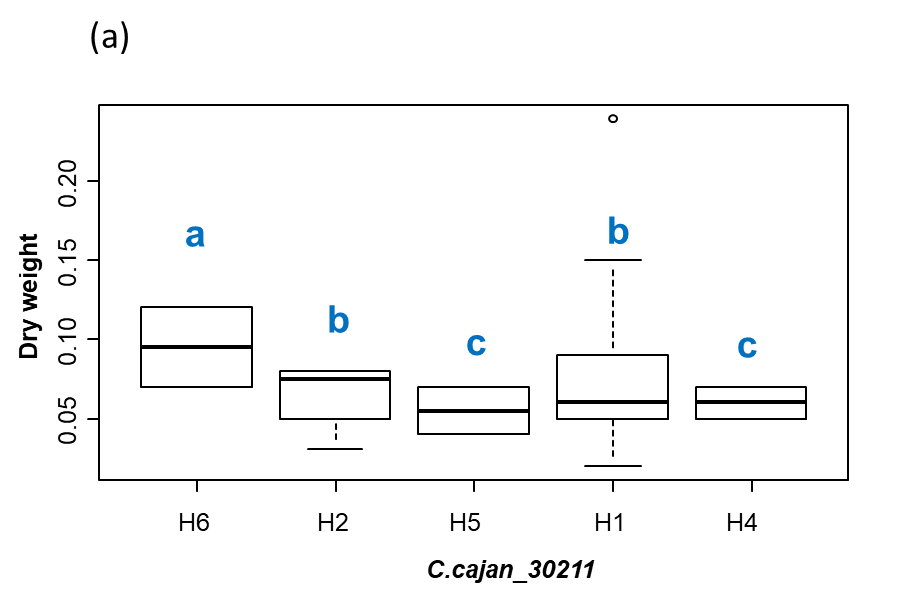


**Figure S4.** Boxplot showing variation in dry weight among 137 pigeonpea accessions with different haplotypes for identification of superior haplotype for plant weight. Lower and upper boxes indicate the 25th and 75th percentile, respectively. The median is depicted by the horizontal line in the box. Duncan’s analysis suggested H6 is superior haplotype for *C.cajan_30211*


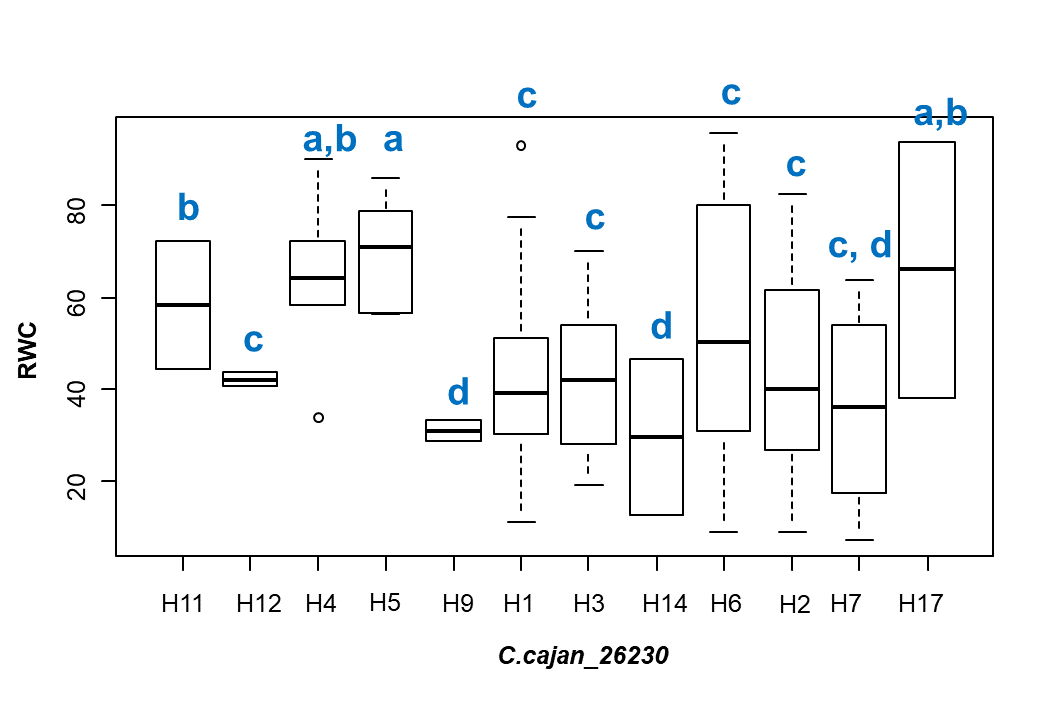


**Figure S5.** Boxplot showing variation in relative water content (RWC) among 137 pigeonpea accessions with different haplotypes for identification of superior haplotype for plant weight. Lower and upper boxes indicate the 25th and 75th percentile, respectively. The median is depicted by the horizontal line in the box. Duncan’s analysis suggested H5 is superior haplotype for *C.cajana_26230.*

| Gene-Id | Uniprot-Id | Protein name |
| --- | --- | --- |
| *C.cajan_26230* | Q9SW11 | U-box domain-containing protein 35 |
| *C.cajan_39705* | Q9SW11 | U-box domain-containing protein 35 |
| *C.cajan_09181* | Q8GZ84 | U-box domain-containing protein 36 |
| *C.cajan_30211* | Q9FKG6 | U-box domain-containing protein 52 |
| *C.cajan_46779* | Q9SIT5 | Cation/H(+) antiporter 15 |
| *C.cajan_08737* | I1JEJ0 | Uncharacterized protein |
| *C.cajan_13768* | Q8LGG8 | Universal stress protein A-like protein |
| *C.cajan_23080* | Q57951 | Universal stress protein |
| *C.cajan_29830* | Q8LGG8 | Universal stress protein A-like protein |
| *C.cajan_33874* | Q8LGG8 | Universal stress protein A-like protein |

**Table S1**. List of genes selected for haplotype analysis

**Table S2**. Haplotype frequency of 10 drought-responsive genes (Dataset in excel)

**Table S3**. Number of unique haplotypes, distribution and frequency range of 10 selected genes in the pigeonpea reference set (292 genotypes) (Dataset in excel)

**Table S4.** Details about 137 *Cajanus* accessions utilized for *Haplo-pheno* analysis

(Dataset in excel)

**Table S5**. Descriptive statistics of 137 *Cajanus* accessions of the targeted drought responsive traits

|  | Minimum | Maximum | Mean | SD | Median | Mode | Kurtosis | Skewness | Standard Error |
| --- | --- | --- | --- | --- | --- | --- | --- | --- | --- |
| Plant weight (PW, g) | 0.11 | 2.17 | 0.87 | 0.33 | 0.84 | 0.84 | 1.08 | 0.53 | 0.03 |
| Shoot length (SL, cm) | 4.75 | 23.50 | 16.03 | 3.63 | 16.33 | 19.00 | 0.83 | -0.74 | 0.31 |
| Root lengt (RL, cm) | 5.00 | 24.33 | 13.05 | 3.39 | 12.67 | 13.00 | 0.94 | 0.66 | 0.29 |
| Fresh weight (FW, g) | 0.03 | 0.68 | 0.19 | 0.11 | 0.17 | 0.12 | 3.58 | 1.50 | 0.01 |
| Turgid weight (TW, g) | 0.03 | 1.28 | 0.32 | 0.18 | 0.30 | 0.15 | 4.60 | 1.44 | 0.02 |
| Dry weight (DW, g) | 0.02 | 0.24 | 0.07 | 0.04 | 0.07 | 0.05 | 3.43 | 1.33 | 0.00 |
| Relative water content (RWC, %) | 7.58 | 98.96 | 47.65 | 21.06 | 45.89 | 33.33 | -0.69 | 0.24 | 1.78 |
|  |  |  |  |  |  |  |  |  |  |
